# Toward a Universal Definition of Physical Frailty Syndrome in Older Adults With Cardiovascular Disease

**DOI:** 10.1016/j.jacadv.2025.102273

**Published:** 2025-10-29

**Authors:** Abdulla A. Damluji, Vincent Chen, Michael G. Nanna

**Affiliations:** a Cardiovascular Center on Aging, Department of Cardiovascular Medicine, Cleveland Clinic Foundation, Cleveland, Ohio, USA; b Section of Cardiovascular Medicine, Department of Medicine, Yale School of Medicine, New Haven, Connecticut, USA

**Keywords:** cardiovascular disease, Cardiovascular-Kidney-Metabolic syndrome, physical frailty, prognosis/prognostic value, risk stratification

**Figure:**
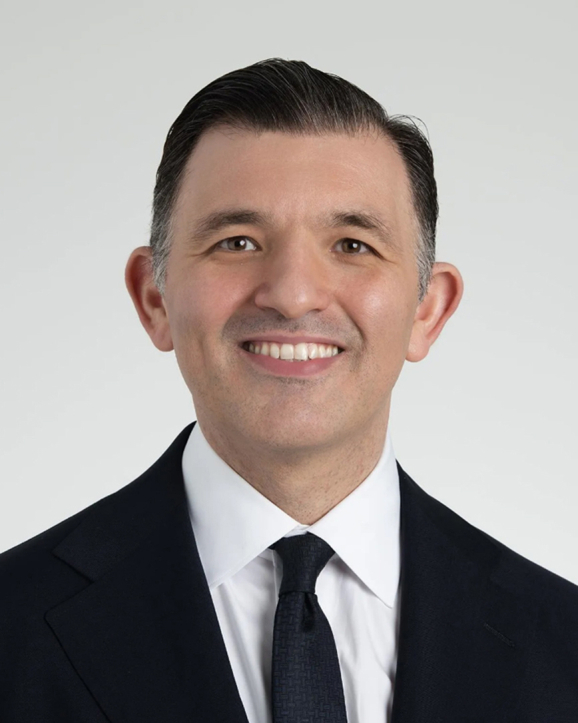

Aging is not a disease, and research is increasingly focused on understanding the aging process by examining biological traits in diverse cohorts of older adults with cardiovascular disease (CVD). Similarly, frailty is not a label but a geriatric condition that reflects underlying physiological vulnerability to stressors across multiple organ systems. Frailty leads to impairment in mobility, cognition, and energy levels for older patients and the risk for developing physical frailty increases with age and the presence of multiple chronic conditions.^1^ Despite the many available instruments to measure frailty reported in the cardiovascular literature, two complementary frameworks exist in research settings:^1^ the Fried Physical Frailty Phenotype evaluates slowness, weakness, weight loss, exhaustion, and low activity with 3 out of 5 of these criteria indicating frailty;^2^ the deficit accumulation approach, which measures health deficits into a continuous frailty index.^1^ Both of these frameworks predict hospitalization, disability, and death in many populations including those with CVD.

The evidence supports that cardiovascular-kidney-metabolic syndrome is a unifying entity to encompass the strong pathophysiological association between adiposity, glycemic dysregulation, hypertension, dyslipidemia, and chronic kidney disease. The American Heart Association provides a framework for identifying patients with Cardiovascular-Kidney-Metabolic (CKM) syndrome in an effort to diagnose early stages of CKM syndrome to prevent progression to overt CVD.^2^ The CKM syndrome staging system ranges from 0, no CKM risk factors; stage 1, excess or dysfunctional adiposity; stage 2, metabolic risk factors and/or moderate to high risk chronic kidney disease; stage 3, subclinical CVD in CKM; and stage 4, established CVD or kidney disease. This classification system provides a common standardized language when referring to cardiometabolic kidney disease. Frailty and CKM syndrome have the same underlying pathophysiological mechanisms including inflammaging, insulin resistance, sarcopenia, endothelial dysfunction, and autonomic dysregulation. Thus, frailty and CKM syndrome coexist and influence each other and the risk for CVD.

The study by Zheng et al^3^ published in this issue of *JACC: Advances* contributes to the literature by identifying frailty as an important risk factor for the development of CVD. While the association of frailty with cardiovascular outcomes is well-established, this study extends the findings to patients with higher CVD risk defined by CKM stages 1 to 3. Among their 3,616 analyzed participants with CKD stages 1 to 3, those with frailty had an 89% increased risk (HR: 1.89; 95% CI: 1.59-2.30) of incident CVD during survey follow-up compared to those without. The authors noted that development of prefrailty or frailty at the year 2 reassessment from the nonfrail (“robust”) state at baseline was associated with a 70% increased risk of CVD (HR: 1.70; 95% CI: 1.38-2.09) compared with those who maintained their nonfrail state. Importantly, those who maintained their nonrobust state from baseline to frailty reassessment more than doubled their risk for incident CVD (HR: 2.35; 95% CI: 1.99-2.78), while improvement or reversion to the prefrail or nonfrail states mitigated the deleterious impact (HR: 1.20; 95% CI: 0.92-1.57) of sustaining the nonrobust state.^3^

A meta-analysis by Kojima et al analyzed 18 cohort studies and provided the foundational evidence for a dose-response association of frailty with clinical outcomes: each 0.01 increase in frailty index conferred a 3.9% increased mortality risk (pooled HR: 1.039; 95% CI: 1.033-1.044).^4^ This relationship has been consistently demonstrated across diverse populations and settings. Data from the United States’ National Health and Aging Trends Study demonstrated similar findings in older adults without established CVD: frailty (as defined by the 5-component Fried Physical Frailty Phenotype) conferred a 77% increased risk of major adverse cardiovascular events over 6 years of follow-up (HR: 1.77; 95% CI: 1.53-2.10).^5^ The present study, then, emphasizes the importance of frailty as a dynamic predictor of cardiovascular morbidity and mortality. Frailty, often seen as a geriatric syndrome, should not be accepted as a static state; rather, efforts to improve the measurement of frailty status at different time intervals by a trained geriatrician or geriatric cardiologist are important because improvement in frailty status over time may attenuate CVD risk and allow for improved overall morbidity burden at the end of life.

Several methodological limitations deserve attention. Most notably, cardiovascular outcomes were ascertained through self-report rather than objective medical records or claims data. This approach introduces potential for misclassification bias, also perhaps exacerbated among those with lower educational attainment and potential for lower health literacy. These also tend to be individuals with worse frailty indices in the study population. This concern is mitigated by a recent analysis by Wang et al from the same CHARLS cohort but with inclusion of a genetic Mendelian randomization analysis, which bolsters the identified relationship between frailty index, change in frailty index, and incident CVD.^6^

The findings from Zheng et al, while not entirely surprising, do have several clinical implications worth considering, particularly in the context of the aging population and the critical need for comprehensive frailty recognition.^3^ While the authors studied a relatively younger cohort (median age: 57 years), the principles of dynamic frailty assessment become increasingly pertinent with advancing age. The established evidence from National Health and Aging Trends Study and other studies of older adults (mean age >75 years) demonstrate that frailty's prognostic impact increases with worse biological age.^7^ This highlights the need for health care systems to develop systematic approaches to identify frailty at index admissions and monitor progression over time, particularly in older adults where the stakes are highest.^8^ The emphasis on repeated measures of frailty is particularly important in older adults, where rapid functional decline can occur over a protracted illness or after a complicated recovery from a procedure.^9^^,^^10^

The use of the CKM characterization helps to describe the heterogeneity of incident CVD risk in older adults, who are a traditionally undercharacterized monolith. An analysis by Zhang et al, also on the CHARLS data set, showed that those with CKM 1 to 2 demonstrated a differential sensitivity to abdominal obesity in progression of frailty than those with CKM 3.^11^ Adjudication of frailty, CKM stage, and obesity not only helps define global CVD risk but also helps tailor interventions to the individual older patient. Each of these variables provides incremental value for risk prediction, particularly in intermediate-risk populations where additional risk markers could influence management decisions.^12^ In older adults with CKM 1 to 3, frailty assessment is even more valuable given the higher baseline risk and greater potential for functional decline.

It must be noted that while the consistency of findings across multiple studies suggests that using a frailty index may be preferable to simpler screening tools, the practical challenges of implementing comprehensive frailty assessment in routine clinical care remain substantial. For older adults, where frailty prevalence is highest, developing streamlined yet comprehensive assessment approaches is particularly critical. Certain screening tools have not been shown to add incremental risk prediction to standard CVD confounders, but there has been a signal for CVD risk especially in those with fewer traditional risk factors, more akin to a CKM 0 phenotype.^13^

The authors should be congratulated for their current analysis on frailty syndrome as a dynamic process in patients with CKM syndrome, but we reiterate that the aging demographic transition worldwide requires a better understanding of the interaction between frailty and CKM status in older adults. Studies specifically examining frailty progression in adults ≥65 years of age across the spectrum of CKM stages could provide insights for geriatric cardiovascular care, where frailty recognition and intervention may have the greatest impact on outcomes and quality of life.^9^ Further studies should also prioritize objective outcome assessment through medical records or claims data rather than self-report, as in the present study. Trials examining whether specific frailty interventions reduce CVD risk are also needed, which are again of great interest in chronologically older adults.

The study by Zheng et al provides incremental evidence supporting the prognostic value of frailty syndrome as a dynamic process in CKM risk staging, building upon established findings from prior analyses. What the field now needs more than any time in the past is a standardized frailty definition and reporting framework endorsed by cardiovascular societies including the American College of Cardiology, American Heart Association, and the European Society of Cardiology. There is significant heterogeneity in phenotypic scores, deficit accumulation indices, clinical scales, single-item tests reflecting the influence of frailty on CVD outcomes. Such heterogeneity complicates clinical decision-making and interpretation of clinical studies. A common standard would specify core domains, cut points, timing of assessment, and minimum reporting criteria and cross validation between instruments. This will also improve integration of frailty assessment in clinical trials and future interventions. Such standardization would facilitate the implementation of frailty into clinical trial eligibility and risk stratification, power to detect differential treatment effects, harmonize risk calculation, and translate into a bedside instrument for clinical practice.

Frailty is not merely a static condition, but a dynamic process with important prognostic implications. The importance of systematically monitoring frailty is amplified in older adults, where early recognition of frailty progression could delay incident CVD and improve outcomes and quality of life. The ever-evolving field of geriatric cardiology reinforces the need for a systematic approach to caring for an aging population that has been misclassified as homogeneous for far too long.^8^

## Funding support and author disclosures

This study was funded in part by a mentored patient-oriented research career development award from the 10.13039/100000050National Heart, Lung, and Blood Institute (K23-HL153771-01). Dr Damluji has received research funding from the Pepper Scholars Program of the Johns Hopkins University Claude D. Pepper Older Americans Independence Center funded by the 10.13039/100000049National Institute on Aging P30-AG021334 and has received mentored patient-oriented research career development award from the 10.13039/100000050National Heart, Lung, and Blood Institute K23-HL153771-01. Dr Nanna has received unrelated current research support from the 10.13039/100005485American College of Cardiology Foundation supported by the George F. and Ann Harris Bellows Foundation, the 10.13039/100006093Patient-Centered Outcomes Research Institute (PCORI), the Yale Claude D. Pepper Older Americans Independence Center (P30AG021342), and the 10.13039/100000049National Institute on Aging (K76AG088428); and has received personal fees from Heartflow, Inc, Merck, and Novo Nordisk. Dr Chen has reported that he has no relationships relevant to the contents of this paper to disclose.
